# Outcomes of microvascular decompression for trigeminal neuralgia: a retrospective analysis from a Mexican high-specialty center

**DOI:** 10.1055/s-0046-1817053

**Published:** 2026-02-27

**Authors:** Iván Eduardo Gonzalez-Gonzalez, Andrés Alberto Moral-Naranjo, Julio César López-Valdés, Óscar Medina-Carrillo, Daniel Alejandro Vega-Moreno, Alejandro Jacob Madrid-Sánchez, Alexis Manuel Portillo-González, Ulises García-González

**Affiliations:** 1 Universidad Nacional Autónoma de México, Facultad de Medicina, División de Estudios de Posgrado, Ciudad de México, Mexico.; 2 Hospital Central Sur de Alta Especialidad Petróleos Mexicanos (PEMEX), Departamento de Neurología y Neurocirugía, Ciudad de México, Mexico.; 3 Universidad Autónoma de Tamaulipas, Facultad de Medicina “Dr. Alberto Romo Caballero”, Departamento de Investigación, Tampico TM Mexico.; 4 Centro Médico del Instituto de Seguridad Social del Estado de México y Municipios (ISSEMYM), Departamento de Neurocirugía Oncológica, San Jerónimo Chicahualco EM, Mexico.; 5 Hospital Central Norte Petróleos Mexicanos (PEMEX), Departamento de Neurocirugía, Ciudad de México, Mexico.

**Keywords:** Trigeminal Neuralgia, Microvascular Decompression Surgery, Treatment Outcome, Nerve Compression Syndromes, Pain Measurement

## Abstract

**Background:**

Trigeminal neuralgia (TN) is a severe craniofacial pain disorder that significantly impacts patient quality of life. Microvascular decompression (MVD) is the sole surgical technique directly addressing the underlying neurovascular conflict, offering durable pain relief. However, data from Latin American populations remain limited.

**Objective:**

To evaluate pain improvement, recurrence, and complications in patients with classic TN undergoing MVD at a Mexican high-specialty center.

**Methods:**

We retrospectively analyzed 61 consecutive classic TN patients who underwent MVD (2010–2023). Their clinical records provided demographic data, pain characteristics, Barrow Neurological Institute Pain Scale (BNI-PS) scores, intraoperative findings, and long-term outcomes. The statistical analyses were performed through the McNemar and Cochran Q tests for longitudinal changes.

**Results:**

The cohort was predominantly composed of women (80.3%; median age: 59 years). Preoperatively, most reported severe pain (BNI-PS score: IV–V). After MVD, 57.4% achieved complete pain relief (BNI-PS score: I) and 13.1% had occasional pain not requiring medication (BNI-PS score: II) at the long-term follow-up. Neurovascular compression was identified in 96.7% of the cases, most frequently by the superior cerebellar artery (49.2%). The overall recurrence rate was of 14.8%, with 8.2% requiring reoperation. Early complications included transient cerebellar syndrome (8.2%) and facial hypoesthesia (11.5%); 1 case of mortality occurred due to hypertensive hemorrhage.

**Conclusion:**

Microvascular decompression is a safe, effective, and durable treatment for classic TN, achieving long-term pain relief in more than 70% of the patients. The present study offers valuable clinical data from a Mexican cohort, reinforcing the efficacy and reproducibility of the procedure in diverse neurosurgical settings.

## INTRODUCTION

Trigeminal neuralgia (TN) is a chronic pain condition involving the fifth cranial nerve, marked by recurrent episodes of sudden, sharp, electric shock-like facial pain. 1 2 3 4 These episodes are often triggered by minimal stimuli, such as chewing, brushing teeth, or touching the face. 1 3 Several studies 5 6 7 reveal a global incidence of 12.6 to 28.9 cases per 100 thousand individuals per year. Other series report a lower incidence, of up to 4.7 per 100 thousand inhabitants. 8 It is more prevalent in female subjects, with a 1.5:1 female-to-male ratio. 1

The pathophysiology of TN appears to be complex and multifactorial. It is now accepted that the mechanism is not solely dependent on a single cause, but rather involves a combination of genetic predisposition, anatomical changes, and neurophysiological factors. This convergence of factors leads to hyperexcitable neuronal states, central sensitization, and widespread neural plasticity changes. This suggests that the pathophysiology of the condition evolves through multiple mechanisms, making it a multifaceted disorder. 9

It is accepted that the most common cause is compression by a nearby blood vessel. 5 While pharmacologic management using agents such as carbamazepine or oxcarbazepine is typically first-line, many patients experience either insufficient pain control or intolerable side effects, requiring surgical intervention. 1 2 3 4

Microvascular decompression (MVD), developed by Peter Jannetta, 10 is the only surgical option that addresses the root cause of TN vascular compression at the root entry zone of the trigeminal nerve. Microvascular decompression has been widely adopted in the neurosurgical practice, and it is considered the gold standard for medically-refractory classic TN. Despite robust evidence supporting its efficacy and safety, there is limited data from Latin American populations. 5 6

The current study aims to evaluate the improvement in pain in Mexican patients with TN undergoing MVD.

## METHODS

A retrospective, observational, single-center study was conducted in the Department of Neurology and Neurosurgery at Hospital Central Sur de Alta Especialidad Petróleos Mexicanos between January 2010 and December 2023.

Convenience sampling of patients was conducted using the code of the International Classification of Diseases, 10th revision (ICD-10) 11 for TN, which is is G50.0. According to the recommendations of the Strengthening the Reporting of Observational Studies in Epidemiology (STROBE) statement, the electronic data from the clinical records of the patients diagnosed with TN were analyzed. The present research adhered to the principles outlined in the Declaration of Helsinki. The institutional Review Board approved the study, granting a waiver for informed consent (under number HCSAE-66-2023). Data confidentiality was guaranteed in accordance with Mexico's General Health Law. 12

### Patient selection

The inclusion criteria were patients diagnosed with classic TN according to the criteria of the beta version of the third edition of the International Classification of Headache Disorders (ICHD-3) 13 ( Table 1 ) who underwent MVD via the conventional retrosigmoid approach; 14 subjects with complete clinical documentation; and those undergoing postoperative follow-up within the institution (for at least 12 months).

**Table 1:** Criteria for trigeminal neuralgia, according to the criteria of the beta version of the third edition of the International Classification of Headache Disorders (ICHD-3 beta) 13

Criteria	Description
A	At least three attacks of unilateral facial pain meeting criteria B and C.
B	Occurring in one or more divisions of the trigeminal nerve, with no radiation beyond the trigeminal distribution.
C	The pain has at least three of the following four characteristics:
	• Recurrent paroxysmal attacks lasting from a fraction of a second to two minutes.
	• Severe intensity.
	• Quality like an electric shock, shooting, stabbing, or sharp pain.
	• Precipitated by innocuous stimuli to the affected side of the face.
D	No clinically-evident neurological deficit.
E	Not better explained by another ICHD-3 diagnosis.

Microvascular decompression was indicated for patients with classic TN refractory to medical therapy (carbamazepine or oxcarbazepine) and with clinical or radiological suspicion of vascular compression.

The exclusion criteria were as follows: patients with secondary TN (such as TN caused by tumors, multiple sclerosis), atypical facial pain, and incomplete records. Patients with a concurrent pain condition, previous interventions outside of the institution, or who did not complete the clinical follow-up were excluded.

### Data collection

The variables analyzed included demographic data, pain semiology (type, side, dermatomal distribution), comorbidities (hypertension, diabetes, smoking), pre- and postoperative scores on the Barrow Neurological Institute Pain Scale (BNI-PS), 15 intraoperative findings (regarding vascular structures), recurrence, and early/late complications.

The results for the research outcome were evaluated based on the BNI-PS scores ( Table 2 ). For the current study, recurrence was defined as return of facial pain with BNI-PS score worsening after initial postoperative improvement.

**Table 2:** Barrow Neurological Institute Pain Scale (BNI-PS) 15

Score	Description
I	No trigeminal pain, no medication.
II	Occasional pain, no medication required.
IIIa	No pain, but continuous medication is required.
IIIb	Occasional pain, but adequately controlled with medication.
IV	Frequent pain, not adequately managed with pain medication.
V	Severe, constant pain, no relief.

All patients underwent preoperative high-resolution brain magnetic resonance imaging (MRI) scans (fast imaging employing steady-state acquisition [FIESTA] sequence). Postoperative MRI scans were performed during the follow-up and repeated when the clinical symptoms suggested recurrence or complication.

Data was collected daily through the electronic clinical file by a first investigator (IEGG) using the ICD-10 codes. 11 In the case of missing data, the electronic system for radiological (MRI FIESTA sequence) studies was reviewed (by AAMN); the ICHD-3 beta criteria 13 ( Table 1 ) were applied by direct clinical evaluation and supported by a retrospective review of medical records cinducted by three investigators. (IEGG, AAMN, and JCLV)

### Statistical analysis

A database was structured by two investigators (IEGG and AAMN) using the Microsoft Excel (Microsoft Corp.) software. The statistical analysis was conducted (by JCLV) using the MedCalc (MedCalc Software Ltd) software. Descriptive statistics were used to summarize categorical variables such as sex, affected side, type of vascular compression, and complications, which were expressed as frequencies and percentages. To compare continuous variables such as age between groups, the Student's *t* -test was employed after verifying normality with the Shapiro–Wilk test. Associations between categorical variables, such as the relationship between sex and affected side, or smoking and recurrence, were evaluated using the Chi-squared (χ ^2^ ) test.

To assess the impact of surgery on clinical outcomes throughout time, we used the BNI-PS, which was dichotomized into two categories for analysis: “good outcome”: patients with a BNI-PS score of I or II (pain-free or with occasional pain not requiring medication); and “poor outcome”: patients with a BNI-PS score of IIIa, IIIb, IV, or V (pain requiring medication or persistent pain).

The McNemar's test was used to compare significant changes in the proportion of patients with a “good outcome” in two paired time points, such as the pre- versus the postoperative state, and the preoperative state versus long-term follow-up.

Finally, to evaluate whether the proportion of patients with a “good outcome” changed significantly across all three time points (pre-, postoperative, and follow-up), the Cochran's Q test was employed. A post-hoc Bonferroni correction was applied for pairwise comparisons to ensure the findings were statistically robust. Values of *p* < 0.05 were considered statistically significant for all analyses.

In addition to the analyses performed with the McNemar's and Cochran's Q tests to evaluate longitudinal changes in outcomes throughout time, we conducted exploratory analyses to assess whether pain descriptors and the site of neurovascular compression were associated with surgical results. Since these comparisons involved categorical variables without repeated measures in the same patients, the χ ^2^ test of independence was employed rather than McNemar's test, which is specifically designed for paired dichotomous data across two time points. This approach enabled us to examine potential associations between baseline clinical or anatomical features and postoperative outcomes.

## RESULTS

**Figure 1:**
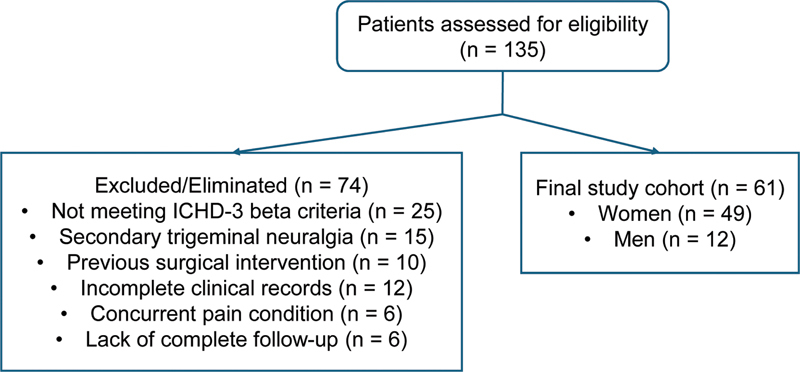
Flowchart for patient selection.

Among the 61 patients included ( Figure 1 ), 80.3% were female individuals. The mean age at the time of surgery was of 59 ± 12.9 (range: 34–89) years. The right side was most commonly affected (63.9%), while 36.1% had left-sided involvement. ( Table 3 ). The duration of pain before surgery was of 95 ± 96 (range: 6–540) months.

**Table 3:** Patient demographic and clinical characteristics

Characteristic	Category	Frequency (n = 61)
Sex: n (%)	Female	49 (80.3%)
	Male	12 (19.7%)
Mean age; range (years)		59 ± 12.9; 34–89
	
Affected side: n (%)	Right	39 (63.9%)
	Left	22 (36.1%)
Comorbidities: n (%)	Hypertension	22 (36.1%)
	Diabetes	6 (9.8%)
	Smoking	15 (24.6%)
Offending vessel: n (%)	Superior cerebellar artery	30 (49.2%)
	Mixed arteriovenous conflict	15 (24.6%)
	Superior petrosal venous complex	8 (13%)
	Other vessels (vertebrobasilar branches)	6 (9.9%)
	No compression identified	2 (3.3%)

The most frequently affected branches were V2 to V3 (47%), followed by V1 to V3 (23%) and isolated V3 involvement (14.8%).

Hypertension was noted in 36.1% of the cases, diabetes, in 9.8%, and a history of smoking, in 24.6%. The most common pain description was “electric shock-like” (50.8%), followed by stabbing (27.9%), burning (13.1%), and hyperesthesia (8.2%). Pain was paroxysmal in 85.2% of the cases and continuous in 14.8%, predominantly affecting the right side (63.9%). The mean follow-up after surgery was of 25.4 ± 31 (range: 12–120) months.

The preoperative BNI-PS scores were as follows: I – 0%; II – 1.6%; IIIa – 11.5%; IIIb – 21.3%; IV – 41.0%; and V – 24.6%. One case was found to be in BNI-PS class II; despite the classification, this patient experienced persistent disabling pain and drug-related adverse effects; therefore, MVD was indicated ( Table 4 ). The postoperative scores improved substantially: I – 57.4%; II – 13.1%; IIIa/IIIb – 9.8%; IV – 14.8%; and V – 1.6%.

**Table 4:** Association of clinical pain characteristics and neurovascular compression site with surgical outcomes after microvascular decompression

Pain descriptor	Good outcome (BNI-PS I–II): n (%)	Poor outcome (BNI-PS III–V): n (%)	Total
Electric shock-like	24 (77.4%)	7 (22.6%)	31
Stabbing	12 (70.6%)	5 (29.4%)	17
Burning	5 (62.5%)	3 (37.5%)	8
Hyperesthesia	2 (40.0%)	3 (60.0%)	5
Total	43 (70.5%)	18 (29.5%)	61
Neurovascular compression site and surgical outcomes			
Compression site	Good outcome (BNI-PS I–II): n (%)	Poor outcome (BNI-PS III–V): n (%)	Total
Superior cerebellar artery	22 (73.3%)	8 (26.7%)	30
Mixed arteriovenous conflict	10 (66.7%)	5 (33.3%)	15
Petrosal venous complex	6 (75.0%)	2 (25.0%)	8
Others	4 (66.7%)	2 (33.3%)	6
No compression	1 (50.0%)	1 (50.0%)	2
Total	43 (70.5%)	18 (29.5%)	61

Abbreviation: BNI-PS, Barrow Neurological Institute Pain Scale.

Intraoperatively, neurovascular compression was identified in 96.7% of the patients. The superior cerebellar artery was the compressive structure most frequently identified (49.2%), followed by mixed arteriovenous compression (24.6%), the petrosal venous complex (13.1%), and others ( Table 4 ). Two patients underwent MVD despite no radiological/intraoperative compression, because they presented with classic refractory TN. Their outcomes did not differ significantly from those of the patients with compression.

**Figure 2:**
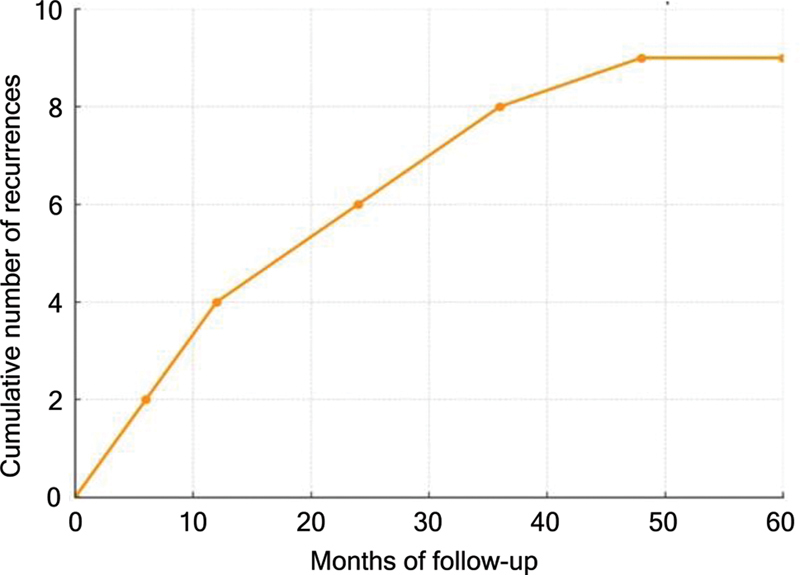
Cumulative number of recurrences throughout the follow-up.

In the immediate postoperative period, 55.7% of the patients improved to BNI-PS II, and, at the long-term follow-up, 57.4% maintained complete pain remission (BNI-PS I), while 13.1% were classified as BNI-PS II. Overall recurrence occurred in 14.8% of the patients, with a mean time until recurrence of 21 ± 14 months ( Figure 2 ). Of these, 71.4% were classified as major recurrences (BNI-PS IV–V) and 28.6%, as minor recurrences (BNI-PS II–IIIb). Surgical reintervention was required in 8.2% of the cases.

The clinical outcomes, measured by the BNI-PS, were analyzed using the McNemar's and Cochran's Q tests to evaluate the impact of MVD throughout time. The McNemar's test revealed a statistically significant improvement in the proportion of patients achieving a “good outcome” (BNI-PS I–II) when comparing the preoperative state to the postoperative period and the long-term follow-up ( *p* < 0.001). This confirms the immediate and sustained effectiveness of the surgery. Furthermore, the Cochran's Q test showed a highly-significant overall change in the proportion of patients with a “good outcome” across all three time points (pre-, postoperative, and follow-up; *p* < 0.001). A subsequent post-hoc analysis with Bonferroni correction confirmed that, while the improvement from the preoperative state was significant, there was no significant difference between the postoperative and long-term follow-up results, indicating that the surgical benefits were durable. The results are shown in Table 5 .

**Table 5:** Statistical analyses

Comparison	Statistical test	Value of the statistical test	Degrees of Freedom (df)	*p* -value
Pre- versus postoperative period ^#^	McNemar's	24.31	1	< 0.001*
Preoperative period versus follow-up ^#^	McNemar's	23.33	1	< 0.001*
Pre-, postoperative period, and follow-up	Cochran's Q	38.35	2	< 0.001*
Sex versus affected side	Chi-squared	0.457	1	0.499
Smoking versus recurrence	Chi-squared	0.509	1	0.476
Mean age by sex	Student's *t* -test	0.536	59	0.594
Time until surgery versus recurrence	Student's *t* -test	1.57	59	0.122

Notes: Comparisons between categorical variables were performed using Chi-squared tests. Longitudinal analyses of pain outcomes were assessed with McNemar's test and Cochran's Q test, as appropriate. Continuous variables were analyzed with the Student's *t* -test. The patients were divided into “good outcome” (score I–II on the Barrow Neurological Institute Pain Scale [BNI-PS]) and “poor outcome” (BNI-PS score: III–V) groups for analysis. ^#^ Post-hoc Bonferroni correction was applied, with statistical significance set at *p* < 0.0167. ^*^ Statistically significant.

An exploratory analysis was performed to evaluate whether clinical pain descriptors or the site of neurovascular compression influenced surgical outcomes. As shown in Table 4 , no significant association was observed between the type of pain descriptor (electric shock-like, stabbing, burning, or hyperesthesia) and the postoperative results (χ ^2^ = 3.20; *p* = 0.36). Similarly, the analysis of neurovascular compression sites demonstrated that the outcomes did not significantly differ according to the vessel involved, including the superior cerebellar artery, mixed arteriovenous conflicts, the petrosal venous complex, or other sites (χ ^2^ = 0.75; *p* = 0.95; Table 4 ). These findings suggest that neither the qualitative characteristics of pain nor the specific vessel implicated in compression reliably predict the likelihood of achieving long-term pain relief after MVD in this cohort.

Postoperative complications were observed in the early and late phases. Early complications included cerebellar syndrome in 32.8% and facial hypoesthesia in 26.2%. One patient died due to posterior fossa hemorrhage related to uncontrolled hypertension. Late complications included hearing loss (4.9%), facial paralysis (3.3%), persistent facial hypoesthesia (3.3%), and persistent cerebellar syndrome (3.3%). Overall, 83.6% of the patients had no late complications. The mean hospital stay was of 3.2 ± 1.1 days, and the return to previous activities was achieved at a mean of 4.9 weeks.

All patients were on medication before surgery. Drugs were tapered off postoperatively in cases of pain remission. At the last follow-up, 72.1% were medication-free.

## DISCUSSION

The present study aimed to confirm the established efficacy of MVD as a definitive treatment for classic TN.

Microvascular decompression remains the surgical treatment of choice for classic TN refractory to pharmacological therapy, with reported long-term success rates higher than 80% and low morbidity in experienced centers. 16 17 Our findings confirm that MVD is a highly-effective intervention for classic TN, which is consistent with success rates reported globally.

The proportion of patients achieving BNI-PS score of I or II (70.5%) aligns with previous series that have reported long-term relief in 60 to 80% of the patients. These outcomes are consistent with those reported in large international series. 17 18

Classic studies by Broggi et al. 18 and Sindou et al. 19 have demonstrated that, even in cases involving venous compression or multiple neurovascular conflicts, MVD can achieve durable pain relief Likewise, Zakrzewska et al. 20 emphasized that MVD remains the only potentially-curative treatment, whereas percutaneous procedures and radiosurgery usually provide only temporary control.

The diagnostic accuracy afforded by the ICHD-3 beta criteria is essential for appropriate patient selection, ensuring improved outcomes. 14 16 21 Focal demyelination due to vascular pulsation against the trigeminal root is widely recognized as the main trigger for paroxysmal pain, supporting the rationale for surgical decompression. 22 Current guidelines by the American Academy of Neurology (AAN) and the European Federation of Neurological Societies (EFNS), as well as a recent European consensus, continue to endorse MVD as the gold standard in classic TN. 23

Regarding recurrence, the present study confirms that reoperation can be successful. In our series, reinterventions were mainly related to dense fibrosis or adhesions rather than Teflon granulomas. Nevertheless, prior reports such as those by Guo et al. 24 describe favorable results after repeated MVD, particularly when a new vascular conflict or granuloma is identified. Crow et al. 25 have also highlighted the value of postoperative high-resolution imaging to detect persistent compression or fibrotic changes, which may guide reintervention.

Anatomical factors play an important role in surgical outcomes. Chai et al. 26 compared inter- and transposition in vertebrobasilar dolichoectasia, reporting superior long-term pain control with vascular transposition and no increase in complications. Similarly, Sánchez-Portocarrero et al. 27 demonstrated that the site of compression (proximal versus distal) and vessel type (artery versus vein) directly influence pain severity and recurrence. In our series, proximal arterial compressions were more often associated with persistent pain, underscoring the need for individualized surgical strategies.

The choice of interposition material is another relevant consideration. González-Llanos and Marín 28 reviewed complications associated with Teflon and Ivalon, noting that both are effective when carefully applied, but may cause granulomas or adhesions if mishandled. 28 29 Although granulomas were not a primary cause of recurrence in the present study, dense fibrosis was observed in some reoperated patients, highlighting the importance of meticulous technique and long-term surveillance. 29

The predominance of right-sided symptoms (63.9%) and the high frequency of superior cerebellar artery involvement (49.2%) mirror trends observed in other studies. While the reasons for right-side predominance remain uncertain, vascular anatomy or embryologic variations may play a role.

The complication rates in our series, particularly cerebellar syndrome (8.2%) and facial hypoesthesia (11.5%), were more frequent than expected, but were largely transient and self-limiting. Xia et al. 30 reported a facial numbness rate of 9.1% after MVD. Additionally, a more recent study 31 with 154 patients using a minimally-invasive approach found a lower overall complication rate of 5.8%, with most neurological complications being transient. The higher rates in the current series may be attributable to factors such as an institutional learning curve and the complexity of the cases.

For clinical outcome assessment, we employed the BNI-PS, 15 which remains widely validated and standardized for TN. Nevertheless, recent tools such as the Facial Pain Score (FPS), validated by Pérez et al., 32 may complement the BNI-PS by providing additional insight into the impacts on quality of life. Wu et al., 33 in a prospective study, confirmed that pain relief and quality of life improve significantly after MVD and remain stable over time, reinforcing the value of structured follow-up.

Minimally-invasive alternatives are also emerging. Velázquez et al. 34 described the asterional approach as a less-invasive alternative that reduces morbidity without compromising visualization of the cerebellopontine angle. Although our series exclusively used the conventional retrosigmoid approach, these techniques warrant future evaluation. Additionally, innovative strategies such as trigeminal nerve separation from vertebrobasilar dolichoectasia using fixation bands or biomedical adhesives, as reported by Yang et al., 35 appear to be safe and effective in complex cases.

Finally, the long-term outcomes remain favorable. Alshawi et al. 36 have demonstrated that more than 80% of the patients maintain durable pain relief with low rates of permanent complications after MVD. Our findings are consistent with these reports, supporting the durability and safety of the procedure.

### Limitations and strenghts

The current study has several limitations. Its retrospective and single-center design introduces potential selection bias. The sample size, although representative, is modest compared to those of international multicenter series. Additionally, the mean follow-up of 28 months is shorter than in some reports extending beyond 5 to 10 years. These factors should be considered when interpreting recurrence rates and long-term efficacy.

One notable strength of the study is the consistent application of theBNI-PS for the pre- and postoperative assessments, providing objective, reproducible outcome metrics.

In conclusion, MVD remains the gold standard surgical treatment for classic TN, especially in patients who fail to respond to medical therapy. This retrospective cohort from a Mexican high-specialty center demonstrates that MVD is effective and safe, with acceptable morbidity and recurrence rates. Our findings support the continued use of MVD in the Latin-American neurosurgical practice and highlight the importance of long-term follow-up and standardized outcome assessment.
